# How can dementia diagnosis and care for Aboriginal and Torres Strait Islander people be improved? Perspectives of healthcare providers providing care in Aboriginal community controlled health services

**DOI:** 10.1186/s12913-021-06647-2

**Published:** 2021-07-16

**Authors:** Jamie Bryant, Natasha Noble, Megan Freund, Jennifer Rumbel, Sandra Eades, Rob Sanson-Fisher, Michael Lowe, Justin Walsh, Leon Piterman, Susan Koch, Claudia Meyer, Elaine Todd

**Affiliations:** 1 School of Medicine and Public Health, University of Newcastle, 2308 Callaghan, NSW Australia; 2 Priority Research Centre for Health Behaviour, University of Newcastle, 2308 Callaghan, NSW Australia; 3 Hunter Medical Research Institute, 2305 New Lambton Heights, NSW Australia; 4 Hunter Medical Research Institute, University Drive, NSW 2308 Callaghan, Australia; 5 Curtin Medical School, Curtin University, 6102 Bentley, WA Australia; 6 Northern Territory Department of Health, 0800 Darwin, NT Australia; 7 Faculty of Health and Medicine, University of Sydney Medical School, University of Sydney, 2006 NSW Sydney, Australia; 8 Faculty of Health and Medicine, University of Newcastle, Callaghan, Australia; 9 Bolton Clarke Research Institute, 3204 Bentleigh, VIC Australia; 10 NSW Consumer Reference Group and Consumer Dementia Research Network, 2113 North Ryde, Dementia Australia, NSW Australia

**Keywords:** Dementia, Alzheimer’s, Aboriginal health, Indigenous health, Aboriginal Community Controlled Health Services

## Abstract

**Background:**

Aboriginal and/or Torres Strait Islander people experience dementia at a rate three to five times higher than the general Australian population. Aboriginal Community Controlled Health Services (ACCHSs) have a critical role to play in recognising symptoms of cognitive impairment, facilitating timely diagnosis of dementia, and managing the impacts of dementia. Little is known about the barriers and enablers to Aboriginal people receiving a timely dementia diagnosis and appropriate care once diagnosed.

This study aims to explore, from the perspective of healthcare providers in the ACCHS sector across urban, regional and remote communities, the barriers and enablers to the provision of dementia diagnosis and care.

**Methods:**

A qualitative study involving semi-structured interviews with staff members working in the ACCHS sector. Aboriginal Health Workers, General Practitioners, nurses, practice or program managers, and Chief Executive Officers were eligible to participate. Consenting ACCHS staff completed a telephone interview administered by a trained interviewer. Interviews were audio-recorded, transcribed, and analysed using qualitative content analysis.

**Results:**

Sixteen staff from 10 ACCHSs participated. Most participants perceived their communities had a limited understanding of dementia. Symptoms of dementia were usually noticed by the GP or another healthcare worker at the ACCHS who had an ongoing relationship with the person. Most participants reported that their service had established referral pathways with either hospital-based geriatricians, geriatricians located with aged care assessment teams, or specialists who visited communities periodically. Key enablers to high quality dementia care included the use of routine health assessments as a mechanism for diagnosis; relationships within communities to support diagnosis and care; community and family relationships; comprehensive and holistic care models; and the use of tailored visual resources to support care. Key barriers to high quality care included: denial and stigma; dementia being perceived as a low priority health condition; limited community awareness and understanding of dementia; lack of staff education and training about dementia; and numerous gaps in service delivery.

**Conclusions:**

Substantially increased investments in supporting best-practice diagnosis and management of dementia in Aboriginal communities are required. ACCHSs have key strengths that should be drawn upon in developing solutions to identified barriers to care.

**Supplementary Information:**

The online version contains supplementary material available at 10.1186/s12913-021-06647-2.

## Background

Aboriginal and/or Torres Strait Islander people (hereafter respectfully referred to as Aboriginal people for brevity) experience dementia at a rate three to five times higher than non-Aboriginal Australians [1–4]. Early onset dementia, defined as dementia that first occurs in a person under the age of 65yrs [5], is also more frequent among Aboriginal people compared to non-Aboriginal people [1, 3]. The proportion of Aboriginal people aged 65 years and over is expected to double from 2016 to 2031, [6] with rates of growth particularly high in older age groups (for example, more than 800 % growth in the 85-plus age group by 2050) [7]. This population aging will likely increase the burden of dementia experienced by Aboriginal people [7].

A timely diagnosis [8, 9] of dementia means individuals and their carers may have more time to discuss their condition with their General Practitioner (GP- i.e. their primary care physician). It helps individuals and their families make decisions about their future while they are cognitively competent, and allows prompt access to medical and psychosocial care [10]. Best practice management of dementia may help to reduce the impact of behavioural and psychosocial symptoms, and ensure individuals can live at home for longer periods [11]. Timely diagnosis and management of dementia also provides an opportunity for individuals, their carers and families to be well supported [12]. As a central provider of primary healthcare for Aboriginal people, Aboriginal Community Controlled Health Services (ACCHSs) have a critical role to play in recognising symptoms of cognitive impairment, facilitating timely diagnosis of dementia, and managing the impacts of dementia [13]. ACCHSs provide culturally appropriate primary health care to around 252,000 people and 2.1 million episodes of care per year [14].

Despite the existence of clinical and practice guidelines for the care of people with dementia, [15, 16] many people presenting with cognitive concerns to a health professional do not routinely receive a timely diagnosis and appropriate management [17, 18]. In the general Australian population, symptoms of dementia are noticed by families an average of 3.1 years before a diagnosis is made. Limited evidence similarly suggests a diagnosis is not made in a timely fashion for Aboriginal people [1]. A study in the Kimberley region of Western Australia by Bradley et al. 2020 indicated only 38 % of Aboriginal people with dementia had been diagnosed by their GP [12]. A range of factors such as stigma associated with dementia, a fear of having to leave Country (i.e. relocation to a metropolitan or regional centre to receive treatment or residential care) [19], a lack of culturally appropriate services, and complex and competing issues facing individuals and communities, have been identified as affecting the timely diagnosis of dementia for Aboriginal Australians [12].

In addition to delays in diagnosis, people may not be provided with best-practice dementia care. A 2010 review by Arkles et al. indicated a general under-representation of Aboriginal people with dementia using government-funded dementia services and other aged care programs [20]. The review also noted a lack of respite services and facilities (i.e. services designed to give carers a break for a limited period of time) [21], in particular for those in remote areas [20]. In a qualitative study in the Kimberley region of Western Australia, Smith et al. 2011 identified a range of obstacles to the successful delivery of dementia services for remote Aboriginal communities [22]. Obstacles included poor communication and cooperation among services, and between individual services and the community; the need for community, caregiver and staff education and training; and limited availability of flexible, culturally safe community care services [22]. These studies suggest there is significant scope to improve the provision of high quality care to Aboriginal people living with dementia.

There are likely to be a range of barriers which affect the quality of dementia care received by Aboriginal Australians. However, very limited prior research has examined barriers and enablers to Aboriginal people receiving a timely dementia diagnosis and appropriate care once diagnosed, particularly from the ACCHS healthcare provider perspective. In their qualitative study, Smith et al. described the unmet needs of individuals with dementia living in remote communities of the Kimberley from the perspective of health service providers and caregivers, and explored perspectives about how care could be improved [22]. However, this study was limited to one remote region of Australia. In addition, the study did not specifically explore dementia diagnosis processes or current processes of care, both of which are critical in order to understand the barriers and enablers to providing quality care.

## Methods

### Aims

 The current study aimed to explore, from the perspective of care providers in the ACCHS sector, and across urban, regional and remote communities, current processes for dementia diagnosis and ongoing care, and barriers and enablers to high quality dementia care.

### Design

A qualitative study involving semi-structured interviews with staff members working in the ACCHS sector. Interviews were analysed using content analysis with modifiable coding systems. The COREQ checklist was used in reporting study findings [23].

### Ethical approval and research oversight

This research was carried out with reference to the principles contained in *Ethical conduct in research with Aboriginal and Torres Strait Islander Peoples and communities: Guidelines for researchers and stakeholders 2018* [24] and ​​​*Keeping research on track II 2018* [25]. Ethics approval was provided by the Aboriginal Health and Medical Research Council of NSW (HREC Reference: 1396/18), University of Newcastle (HREC Reference: H-2018-0362), Central Australian Human Research Ethics Committee (CAHREC Reference: CA-18-3270), Menzies School of Health Research (HREC Reference: 18-3255) and Aboriginal Health Council of South Australia (AHREC Reference: 04-18-794). A study Working Group oversaw all aspects of the research. The Working Group consisted of Aboriginal and non-Aboriginal members with expertise in a range of relevant fields including dementia, Aboriginals health, health behaviour and primary care.

### Sample

 Aboriginal Health Workers or Practitioners (AHWs or AHPs), General Practitioners (GPs), nurses, practice or program managers, and Chief Executive Officers (CEOs) employed in an ACCHS on a part-time or full-time basis were eligible to participate. Multiple staff from the same service were eligible to take part.

### Recruitment

#### Two approaches to recruitment were used

##### (1) Recruitment via ACCHSs

An alphabetical list of all Australian ACCHSs was developed from an online search. The list of ACCHSs was randomised using a randomly generated number sequence and sites were then approached in order and invited to participate. An email and brief overview of the study was sent from the study Principal Investigator (RSF) to the Chief Executive Officer (CEO) of selected ACCHSs, inviting the service to participate in the research. Follow up phone calls were made one to two weeks later. Services which did not respond to the email or phone calls received a reminder email after approximately one month, and another follow up phone call. CEOs who agreed for their service to take part were asked to sign and return an organisational consent form. Following consent, CEOs were asked to forward a study information statement to all eligible staff within the service, inviting them to participate in a telephone interview. Interested staff were asked to either indicate their interest in participating directly to the research team via email or a free call telephone number, or provide verbal consent to their CEO for their contact details to be provided to the research team.

##### (2) Recruitment via staff organisations

The National Aboriginal and Torres Strait Islander Health Worker Association, the Congress of Aboriginal and Torres Strait Islander Nurses and Midwives, and the Australian Indigenous Doctors’ Association provided information about the study to potentially eligible participants, either through sending an email about the study to their members, and/or including information (e.g. a study flyer) about the study in their newsletters or on their websites. Eligible staff were asked to contact the research team via email or a free call telephone number.

 Staff recruited via either method who indicated an interest in participating in an interview were contacted by the research team to confirm their eligibility and schedule a time to conduct the interview.

### Data collection

Interviews followed a semi-structured interview guide that was developed via an iterative process utilising a review of the literature, input from the study Working Group, and feedback from representatives of ACCHSs. The final interview guide (Appendix A) covered the following domains: awareness and understanding of dementia in the community; processes for diagnosis and communication of a diagnosis; processes for providing care and support for those diagnosed with dementia and their families; and barriers and enablers experienced in assessing, diagnosing, and managing dementia. Participants were sent a copy of the interview guide prior to the interview.

Consenting ACCHS staff participated in a single telephone interview administered by a trained interviewer (JB: PhD) over the period from August 2019 to March 2020. The female interviewer is an NHMRC-ARC Dementia Research Development Fellow with experience in Aboriginal health, dementia, and qualitative research. An Aboriginal member of the research team was also present for interviews where possible (JR). The interviewer presented a brief rationale for the study before beginning the interview and participants were then asked to provide verbal informed consent. All interviews except one took place via telephone, with a single interviewee. One interview was conducted face-to-face with two interviewees present. All interviewees were located in their workplaces during the interviews. Interviews were audio-recorded with participant consent and field notes were taken during the interviews. Recruitment continued until the interviewer determined that data saturation was achieved. Services (for recruitment via ACCHSs) or participants (for recruitment via staff organisations) were provided with a $50 gift card as acknowledgement for their contribution to the project.

### Analysis

Audio recordings were transcribed for analysis. Data were analysed using Nvivo software (www.qsrinternational.com), using qualitative content analysis with modifiable coding systems [26]. Three authors undertook data coding (JB, NN, JR) with cross-coding checked by one author (JB). Themes were based on the domains included in the interview guide, with additional codes derived from the data. To ensure credibility of the data, member checking was used to ensure findings accurately reflected participant voices, and to allow participants the opportunity to confirm and/or modify interpretations of data. To facilitate this all participants were provided with a written copy of the study results and interpretation, and asked to provide written and/or verbal feedback on the analysis. Revisions were made based on feedback received. A summary of the final study findings were provided to participating services and individuals.

## Results

A total of 42 services were directly approached and invited to participate. Eleven services declined to participate, 23 did not respond to the invitation or reminders, and eight consented and had staff willing to participate (*n* = 12 participants). Staff from two services (*n* = 4 participants) responded to direct advertising of the study. In total, 16 staff from 10 ACCHSs completed a qualitative telephone interview. Interviews ranged in duration from 12 to 56 min. Table 1 summarises the characteristics of study participants. The amount of time that participants had worked in their current roles ranged from eight months to seven years, with the majority of participants having been in their role for two or more years. No participants reported having specialist training in dementia.

**Table 1:** Demographic characteristics of study participants

Demographic Characteristics		N (%)
**Aboriginal and/or Torres Strait Islander**	Yes No	9 (56 %) 7 (44 %)
**Sex**	Male Female	3 (19 %) 13 (81 %)
**State**	NSW NT QLD	12 (75 %) 2 (12.5 %) 2 (12.5 %)
**Position**	Aboriginal Health Worker General Practitioner Manager/ Director^a^ Registered Nurse Senior Medical Officer Other^b^	1 (6 %) 2 (12.5 %) 6 (37.5 %) 2 (12.5 %) 1 (6 %) 4 (25 %)

^a^Including Practice, Clinical Services, Medical Services, Aged and Community Care, and National Disability Insurance Scheme Managers/Directors; ^b^Other positions included Support Workers and Care and Program Coordinators

 The main themes which emerged from the qualitative interviews were related to: the language used to talk about dementia; dementia diagnosis; care following diagnosis of dementia; enablers to high quality dementia care; and barriers to high quality dementia care. Illustrative quotes for the first three themes are presented in Table 2. Quotes for the remaining themes are presented in text.

### 1. Language used to talk about dementia

The word ‘dementia’ was not often used in communities to describe symptoms or disease. Instead, dementia was variously described as memory problems, being forgetful, going nuts or going mad, having mental problems, or using unique community slang words for being forgetful.

### 2. Dementia diagnosis

Most participants noted that very few people in ACCHSs had been formally diagnosed with dementia. Estimates of the number of patients with a dementia diagnosis ranged from just one up to ‘ten to fifteen’, with most participants reporting less than five people per service with a formal diagnosis. However, participants noted that dementia was probably under-recognised, and that the number of people with dementia was likely to be higher than medical records indicated.

Symptoms of dementia were usually recognised by the GP or another healthcare worker at the ACCHS who had an ongoing relationship with the person. Most participants agreed that few patients or their families identify symptoms; while they might seek medical advice for behaviours such as being confused, forgetting to take medications, aggressive behaviours, or self-neglect, dementia was not suspected or recognised as a potential cause of presenting symptoms. However, families were seen as central to the process of diagnosis, with carer and family reports used to facilitate an accurate diagnosis both with the initial assessment conducted by the GP, and at appointments with specialists.

**Table 2:** Thematic table with illustrative quotes for themes

**Global theme**	**Illustrative quotes **
**Language used to talk about dementia**	· *They'll just say it's about memory problems or they're not the same as they were before. I don't hear the word dementia being used a lot by people… (13)* · *It's not necessarily labelled in that sort of language if you get me…. It's just oh so and so's getting a bit old or that they're just forgetting things a bit…. I suppose that you could say that the symptoms are recognised and supported in community, but in terms of actually recognising that dementia or that could be dementia, that that hasn't been - it hasn't really been talked about like that (15)* · *Not all the time. No. No. They very rarely use that particular word [dementia]. They'll say, oh, that one’s a bit dookun or a bit… that one's a bit booky. You sort of look and see, yeah, they are a bit booky today. Some will just say, oh, that one's forgetful. (5)*
**Dementia diagnosis **	· Few people in ACCHSs have been formally diagnosed with dementia:* The client that we have that is formally diagnosed is just one client that's formally diagnosed, but we do have other clients that we're suspecting and we're pushing through with the GPs to do formal assessments, so that the geriatricians can do formal assessments. (4)* · Families as central to the diagnosis process: *When you’re booking them in for the next check-up, you’d invite - ask them to consider bringing a family member with them. That’s where you do it… a little bit more collateral information... (11)*Recognition of dementia symptoms: *Usually it's not flagged. Usually either myself or one of the nurses notice something, some behaviour or some mental change in a client we've known for a long time - well not a long time but known for a while. There seems to be some change…- it's very rare that the community would report that. They may report some behaviours, some irritating or annoying behaviours, that sometimes come to light but mostly I think it's more the clinicians picking it up in the course of our work. (3); I guess we never get self-identification. There might occasionally have people come in going my elderly relative isn't functioning very well and I want you to check him out. That doesn't seem to happen. It's more picked up incidentally. (14)* · Screening tools for dementia: *It's just an MMSE and we do start doing that for all of our clients in their health check but not until they turn 65. (1)*
**Care following a diagnosis of dementia**	· Referral to a geriatrician: *[T]he local geriatrician at the [x] Hospital start doing about quarterly memory and geriatric clinics initially under a research program. Now he has just continued them and that has actually made a big difference because there's lots more formal diagnoses now, rather than that oh well we know Uncle Arthur's not quite all there but he's okay, that kind of thing. (14)* · Geriatrician access issues: *We can refer people in - again, depending on how advanced a person is, that involves a plane trip in to see the specialist so not all of our dementia patients go into that clinic. (3)* · Management of dementia patients by ACCHSs: *This is the ideal avenue, we would then refer the patient to our health worker, who would follow up with any feedback the patient has after they’ve seen us. Whether they’ve got any further questions, whether other family members might need some education. Then usually there’s a reminder to review that patient, depending on each case, three months later for instance. Or monthly, or yeah. (11)*
**Enablers to high quality dementia care **	· Routine Health Assessments as a mechanism for diagnosis: *[T]he 715 Aboriginal and Torres Strait Islander health check. It’s actually an item number you bill through Medicare. That comes up due every nine and a half months. When you complete it, it actually comes up with an automatic reminder on our system that the patient is due. We can actually send them reminder letters or text messages to remind them that their health check is due. (11)* · Relationships within communities to support diagnosis and care: A*wareness of our people. We know our people… …Yeah. So it's just building that bridge and connection and making them feel safe and trust and helping them to be aware and know of these illnesses, that we are there to help them, not to lock them away. Yeah. (13)* · Community and family: *The best way really I think at the moment is that they shuffle that person [with dementia] around within the community to different family members - that’s the way they handle it most… …But as long as they on their Country and in familiar environments, their regular community or people they know who speak the language - his language - then that's the least traumatic I think. (3)* · Comprehensive, multidisciplinary, and holistic care models:* So we - within the service itself, we've got psychologists and we've got counsellors and we've got a social emotional wellbeing team and we've got our Aboriginal health workers, so we try and support them and keep them home as long as possible. (5)* · The use of tailored visual resources to support care:* We try and do everything here visually, that I try and have visual supports for people. So for my staff, for the clients, the families, because it's how they understand it. I've got some families here who are very well educated and so I communicate differently with them. So I could give them written information and they would be able to read it and understand it (2)*
**Barriers to high quality dementia care**	·Limited community awareness and understanding of dementia:*There isn't self-recognition and there certainly is less family recognition that there may be an issue that is potentially treatable or requires extra services. (14)* ·Denial and stigma:* Yeah, because it's not treatable so it's a terminal condition, for want of a better description. I tend to find that's usually the biggest barrier and then the second biggest barrier is - like one is actually getting them to go to the doctors and being diagnosed, and then actually accepting that they have then that diagnosis. Because people don't want to and it's not them and they are only silly sometimes, you know? People will pass it off for a long time. (10)* · Dementia perceived as a low priority health condition:*I think it's easy to just gloss over memory issues and certainly, clients at [service] present with a whole range of complex issues and usually competing priorities. I think memory is probably one of those things that are put to the bottom of the list and we often don't get to it, whether that's consciously or subconsciously. I think it's usually more pressing needs until the family presents in crisis sometimes. (1)* · Community literacy and the need for accessible and culturally appropriate resources:* I think resources or tools that they can understand. Things that are more appropriate for them, to be honest. There's no point printing out something that you would give to a middle-class white person, here you go, here's some reading material. No, if you're going to - teaching needs to be about presenting it in a way that they can understand it and that's lacking. It's lacking across a lot of areas for Aboriginal communities, particularly around aged care. (2)* · Lack of staff knowledge, education and training about dementia: *Having a documented coordinator would be really useful I think, just because as I said, at the moment we've got a de facto person just simply because the other work she does… …I'll give you another example where things are better - and that is that we have regular endocrinology [unclear] which are outreach from the hospital in our service and have done for many, many years. The diabetes educator coordinates that and so it runs like clockwork. People don't get left behind; they don't get forgotten. They get rung a day in advance just to make sure that they're still coming (14) * · Gaps in service delivery: *The other thing - the biggest problem is distance. So sometimes respite care - so for people to go into respite care to give the family a bit of a break means travelling 400 kilometres into [town name] and then put into a place which they don't recognise and it's foreign. You know what that does - dementia people clearly become disorientated and become quite distressed in that sort of situation so that is a problem. It is a problem more for families to be able to get some sort of respite. (3)*

Several participants also described dementia being identified as part of routine 715 Health Assessments (a Medicare Benefits Scheme item which provides reimbursement to GPs for the conduct of an annual health check for Aboriginal peoples of all ages, designed to support physical, social and emotional wellbeing) [27]. A variety of screening tools, most often the Mini Mental State Exam (MMSE) or the Kimberley Indigenous Cognitive Assessment (KICA), were incorporated into health assessments for people aged 55 years or over to screen for cognitive impairment. The General Practitioner Assessment of Cognition (GPCOG), Montreal Cognitive Assessment (MoCA) Test and Rowland Universal Dementia Assessment Scale (RUDAS) were other tools that were sometimes used.

### 3. Care following a diagnosis of dementia

Following recognition of possible cognitive impairment, people were usually referred to a geriatrician for diagnosis. Most participants reported that their service had established referral pathways with either hospital-based geriatricians, geriatricians located with aged care assessment teams, or visiting specialists who visited communities periodically. The geriatrician was reported to be the person who usually communicated the diagnosis to the person and their family. For some remote communities, referral to a geriatrician was not always possible due to access issues. Following diagnosis, most people were referred back to their usual GP or ACCHS for further education, support and ongoing management. ACCHSs were perceived as well placed to deliver culturally appropriate dementia related care through their community outreach and other community activities, such as ‘pamper days’ and elder’s groups.

### 4. Enablers to high quality dementia care

Participants identified a number of enablers to high quality care for people with dementia.

#### 1. Routine Health Assessments as a mechanism for diagnosis

A number of respondents mentioned the benefits of 715 Health Assessments and reminder systems for enabling identification of cognitive impairment or dementia. ACCHSs were noted to have longer appointment times for completion of Health Assessments, as well as healthcare providers able to deliver culturally appropriate assessments. Several respondents noted that the use of dementia screening tools is becoming a routine part of health assessments.

#### 2. Relationships within communities to support diagnosis and care

The strong and familiar relationships built between healthcare workers and their communities is a key strength in the delivery of dementia diagnosis and care. ACCHS staff feel in a unique position to work with the community because they tend to be part of the community, know the local language, and have established trust with people over time. Respondents mentioned the importance of these relationships in allowing people to feel safe, and allowing staff to raise sensitive issues like dementia. The opportunity to talk about dementia as part of community outreach clinics, rather than within the ACCHS, and strong relationships with patient’s families were perceived as supporting dementia diagnosis and care.

#### 3. Community and family

The local Aboriginal community and family represents a core strength in dementia care. The community generally looks out for and supports the person with dementia, and families tend to share the care of family members with dementia. The importance of a person with dementia being able to remain on Country and in a familiar environment was emphasised, because the worst thing is for people to die off-Country [2].

#### 4. Comprehensive, multidisciplinary, and holistic care models

The comprehensive and holistic model of care that ACCHSs offer was seen as a critical to supporting people with dementia and their families. Multidisciplinary assistance provided through ACCHSs included supporting social and emotional wellbeing by providing counselling, psychology, and health promotion services, alongside clinical services. Several respondents mentioned the importance and benefits of providing transport services to improve access to services and referrals. One participant mentioned that their service was focusing on trauma-informed care, given the links between childhood adversity and the development of dementia.

#### 5. The use of tailored visual resources to support care

The use of tailored, pictorial, visual or photographic resources for both healthcare staff and people with dementia was seen to be extremely beneficial in the provision of dementia information and care. Visual resources were needed to overcome any issues with literacy and numeracy.

### 5. Barriers to high quality dementia care

Six main barriers to high quality care for people with dementia were identified.

#### 1. Limited community awareness and understanding of dementia

Most participants perceived their communities had limited understanding of dementia. The signs and symptoms were often not noticed by families, and if they were, they were thought to be natural forgetfulness or memory problems associated with age, rather than a disease that required treatment Several respondents identified the need for culturally specific information and education resources, day talks, or community education workshops to raise community awareness about dementia and the services and supports available. Greater awareness that people with dementia can remain in the community, live with dignity, and receive medical, psychosocial and practical help were emphasised.

#### 2. Denial and stigma

Participants reiterated issues of denial, stigma, and fear of losing independence as key barriers in the process of providing dementia diagnosis and care in Aboriginal communities. People and families may avoid seeking care for signs of dementia, and were reluctant to accept a diagnosis of dementia or a referral for further assessment or treatment.

Mistrust by Aboriginal people and communities of existing and/or mainstream services was mentioned by a number of respondents as a barrier to seeking or accepting dementia care, particularly in the context of ongoing paternalistic policies of colonisation and the Stolen Generations [28]. One participant suggested that racism and stereotyping prevent early recognition and diagnosis of dementia in Aboriginal communities where healthcare providers dismiss or deny Aboriginal people’s healthcare needs.

#### 3. Dementia perceived as a low priority health condition

Numerous participants indicated that dementia is generally not a high priority for the community, or for ACCHSs, as people and clinicians are busy dealing with many other acute and chronic conditions. There was a perception that dementia was not as important as other health conditions, not a ‘legitimate’ disease or concern, compared to diabetes or heart disease for example. Communities and families were also reported to be facing many other pressing social issues, such as poverty, a lack of transport, and mental health and substance use issues, which make it difficult for people to access care. Another issue that was raised was potential symptoms of dementia being overlooked or dismissed as being due to ‘repercussions of lifestyle’, such as the effects of using alcohol or marijuana, by both communities and families, and sometimes also by healthcare staff. Other competing priorities, such as caring responsibilities, might also prevent someone from seeking or accessing care.

#### 4. Community literacy and the need for accessible and culturally appropriate resources

Many respondents noted the need for culturally appropriate resources which are accessible and tailored to various educational levels within communities, in particular the need for simple materials which are predominantly visual rather than text based. The existing resources were generally perceived as inappropriate. There was also a perceived need for better awareness about existing dementia resources.

#### 5. Lack of staff knowledge, education and training about dementia

Limited understanding about dementia among staff, lack of confidence in determining a dementia diagnosis in primary care, and a lack of dementia-specific education and training, were reported barriers to both diagnosis and high quality care. Problems associated with a lack of time during appointments, lack of staff, and a high turnover of staff were also mentioned. One respondent suggested the need for additional funding for a coordinator to oversee dementia care and management, similar to what occurs for other diseases such as diabetes, to ensure that people and families received appropriate and ongoing care and follow up.

#### 6. Gaps in service delivery

One of the most frequently mentioned barriers to effective dementia care was related to gaps in services or a general lack of services for Aboriginal people with dementia. Other issues were related to problems accessing care, such as long distances to travel, financial barriers, and a lack of clear pathways of care for people diagnosed with dementia. Respondents indicated that pathways were often not available to provide appropriate clinical referral for patients, or appropriate “*programs around support, about education, around diagnosis, and around community awareness” (11). *Respondents reported long waits for services, and that the current funding structure for supported aged care services often resulted in those eligible for services not getting access to the support they need.Respondents also reported a lack of culturally appropriate local services for people with dementia and their carers, and no access to “*culturally friendly nursing homes” (1). *Many services that were available (such as respite) were provided off country, and often involved significant travel. Problems accessing care for financial reasons were also frequently mentioned. Respondents reported that there was a “*financial barrier of being able to get care and being able to accept care in the home” (10)* for many Aboriginal patients. 

## Discussion

This qualitative study provides a unique perspective on the barriers and enablers to the provision of dementia diagnosis and care for Aboriginal people receiving care from ACCHSs in Australia.

ACCHS staff perceived that dementia was under-recognised and under-diagnosed for Aboriginal patients attending ACCHSs. Barriers to a timely diagnosis for Aboriginal people were reported to include a combination of limited community awareness about dementia, denial and stigma, competing priorities for both the community and health services, and challenges for healthcare staff related to lack of confidence, education, and training. Although many of these barriers to diagnosis may apply generally to older people with dementia, [29] the unique perspective and context for Aboriginal people adds some significant points of difference. For example, additional barriers for Aboriginal people include traditional beliefs about dementia, a mistrust of mainstream services based on historical and current treatment, a lack of culturally appropriate services, language barriers, and fear of being moved off Country if diagnosed. Similar findings have been reported for Aboriginal people living rural and remote communities [7, 30, 31].

Specific efforts are needed to raise Aboriginal community awareness about the signs and symptoms of dementia, to reduce stigma and mistrust, and to support better diagnosis and management.

While some resources for Aboriginal communities are available from organisations such as Dementia Australia, many participants were not aware of and did not make use of them. In addition, these resources are heavily text based and therefore unlikely to be suitable for use in many communities. A video and flip chart resource called ‘Looking out for Dementia’ has been developed in English and three Indigenous languages to inform Indigenous people in remote communities of Northern Territory about dementia [32]. An evaluation of the resource indicated it was effective in raising community awareness. There is significant scope for additional work to develop, adapt and implement resources to increase awareness of dementia in Aboriginal communities other than in remote communities.

A key enabler of the identification of possible cognitive impairment and a subsequent diagnosis of dementia was the use annual health assessments for Aboriginal patients (e.g. Medicare 715 Health Assessments). ACCHSs as well as mainstream GP practices receive a $218.90 reimbursement from the government for each health assessment completed. ACCHSs often link a reminder system for patients within their electronic medical record to encourage clients to attend on an annual basis. However, currently there is no specific requirement in the 715 Health Assessment to assess cognition for those under the age of 55 years, and no specific guidance on the best standardised tools to assess cognition for those patients aged 55 years and over [33]. In addition, 715 Health Assessments remain underutilised, with approximately 57.7 % of Aboriginal males and 56.5 % of Aboriginal females aged 55–64 having had an assessment in the previous two years [34].

Organisational barriers and facilitators regarding ACCHSs and the wider health system were also identified. ACCHSs have a holistic understanding of health that is often not recognised by western approaches to healthcare that can enhance dementia care [30]. Our findings suggest that multi-disciplinary and multi-agency collaborations and care pathways have been developed in many communities to support the diagnosis and management of dementia, and that ACCHSs are well placed to provide this care given ACCHS staff are well known and trusted in their communities. However, participants identified that ACCHS staff require additional training, and resources to support their dementia work, and to address specific gaps identified in service delivery. Many barriers are not unique to Aboriginal communities such difficulties in accessing specialists common for people living in rural and remote Australia [35]. Care provide to Aboriginal people with dementia would be strengthened by supporting the development of well-defined, structured, and supported pathways for care following a diagnosis of dementia, and the provision of more culturally appropriate local services including residential care and respite services.

A key strength of dementia care was the critical role played by community and family in supporting people with dementia to stay on Country and in familiar surroundings. While this is a key strength in dementia care for Aboriginal people, it is critical that appropriate community supports are available to assist families to provide such care, particularly given evidence that Aboriginal people may not seek home care assistance until it is unavoidable [31]. Linking older Aboriginal people with a diagnosis with dementia should be a focus of efforts to address the nationally recognised delays in older Australians receiving government supported aged care services [36]. There should also be a focus on ensuring these aged care services are available on Country and provide culturally appropriate care.

### Strengths and Limitations

The findings of this study are limited by the lack of representation from ACCHSs across all states and territories, limiting the generalisation of the findings. In addition, among the mix of participants interviewed (e.g. GPs/Senior Medical Officers, Managers/ Directors, AHWs, nurses etc.), some staff had more direct experience in providing dementia diagnosis and care compared to others, and were better placed to discuss specific barriers in relation to these processes. However, the mix of participants provided a broad spectrum of reflections on the experiences of dementia in Aboriginal communities and within ACCHSs. Key strengths of the research include the qualitative methodology that allowed for an in-depth exploration of the perspectives of key ACCHS healthcare providers. The validity of data and analysis was ensured through accounting of contradictory evidence in the analysis to guard against researcher bias. Respondent validation was also used to allow participants the opportunity of contributing to the analysis by providing feedback on the researchers’ interpretations of their responses. Aboriginal participants and research team members were key to data interpretation.

## Conclusions

There is a need to actively raise community awareness around dementia, address issues of stigma and denial, and provide culturally appropriate and well-designed educational resources for Aboriginal communities and ACCHS staff. Substantially increased investments in supporting the management of dementia in Aboriginal communities are also required, given the unique geographic, cultural, policy, and practice challenges and opportunities. Given their essential role in delivering care to Aboriginal people, it is crucial that ACCHSs have a role in developing solutions including a voice at the policy level to ensure that the structures and organisations that support older people with dementia in communities, such as Dementia Australia and My Aged Care, provide culturally appropriate care. Future research should partner with the ACCHS sector to explore approaches to promoting understanding and reducing stigma around dementia, as well as implement and test culturally responsive programs to reduce dementia risk and enhance the care of Aboriginal people living with dementia and their families [9, 12].

## Supplementary Information

**Additional file 1.**

## Data Availability

The data generated and analysed during the current study are not publicly available due to participant confidentiality but may be available from the corresponding author on reasonable request and with appropriate approvals.

## Abbreviations

ACCHS: Aboriginal Community Controlled Health Service.
GP: General Practitioner
CEO: Chief Executive Officer
MMSE: Mini Mental State Exam
KICA: Kimberley Indigenous Cognitive Assessment
GP-COG: General Practitioner Assessment of Cognition
MoCA: Montreal Cognitive Assessment
RUDAS: Rowland Universal Dementia Assessment Scale
