# Supplementary material for: How can dementia diagnosis and care for Aboriginal and Torres Strait Islander people be improved? Perspectives of healthcare providers providing care in Aboriginal community controlled health services
Source: BMC Health Serv Res. 2021 Jul 16;21:699. doi: 10.1186/s12913-021-06647-2 (PMC8283853; doi:10.1186/s12913-021-06647-2)
Supplement: Supplementary file 1 — Additional file 1. [file 12913_2021_6647_MOESM1_ESM.docx]

1. **How aware do you think your patients and the local Aboriginal community are generally about dementia?**
2. **How is possible cognitive impairment or dementia usually flagged or first raised at your service?**
3. **How are patients usually diagnosed with dementia in your service?**
4. **How are patients and their support person(s) usually informed about a diagnosis of dementia?**
5. **Following a diagnosis of dementia, what kind of care do dementia patients receive? What are the current processes of caring for people with dementia within your service?**

**6. What kind of support does your service provide specifically for support person(s) of people with dementia?**

1. **Does your service currently use any guidelines or recommendations for the care of people with dementia?**
2. **Is there someone who acts as a care coordinator for patients with dementia and their support person(s) within your service?**
3. **Does your ACCHS have any other strategies in place to guide the care of patients diagnosed with dementia and their support person(s)?**
4. **What do you believe are the main barriers to the diagnosis and care for people with dementia and their support person(s) within your community?**
5. **What enablers do you think could help to overcome these barriers to providing care for people with dementia and their support person(s)?**
6. **Do you have any other comments or thoughts not covered so far about how your service could improve dementia care for patients and their support person(s)?**
